# Supplementary material for: Liver-target nanotechnology facilitates berberine to ameliorate cardio-metabolic diseases
Source: Nat Commun. 2019 Apr 30;10:1981. doi: 10.1038/s41467-019-09852-0 (PMC6491597; doi:10.1038/s41467-019-09852-0)
Supplement: Supplementary file 1 — Supplementary Information [file 41467_2019_9852_MOESM1_ESM.pdf]

Supplementary Information

Liver-target Nanotechnology Facilitates Berberine to Ameliorate Cardio-metabolic Diseases

Hui-Hui Guo et al.

Supplementary Figures

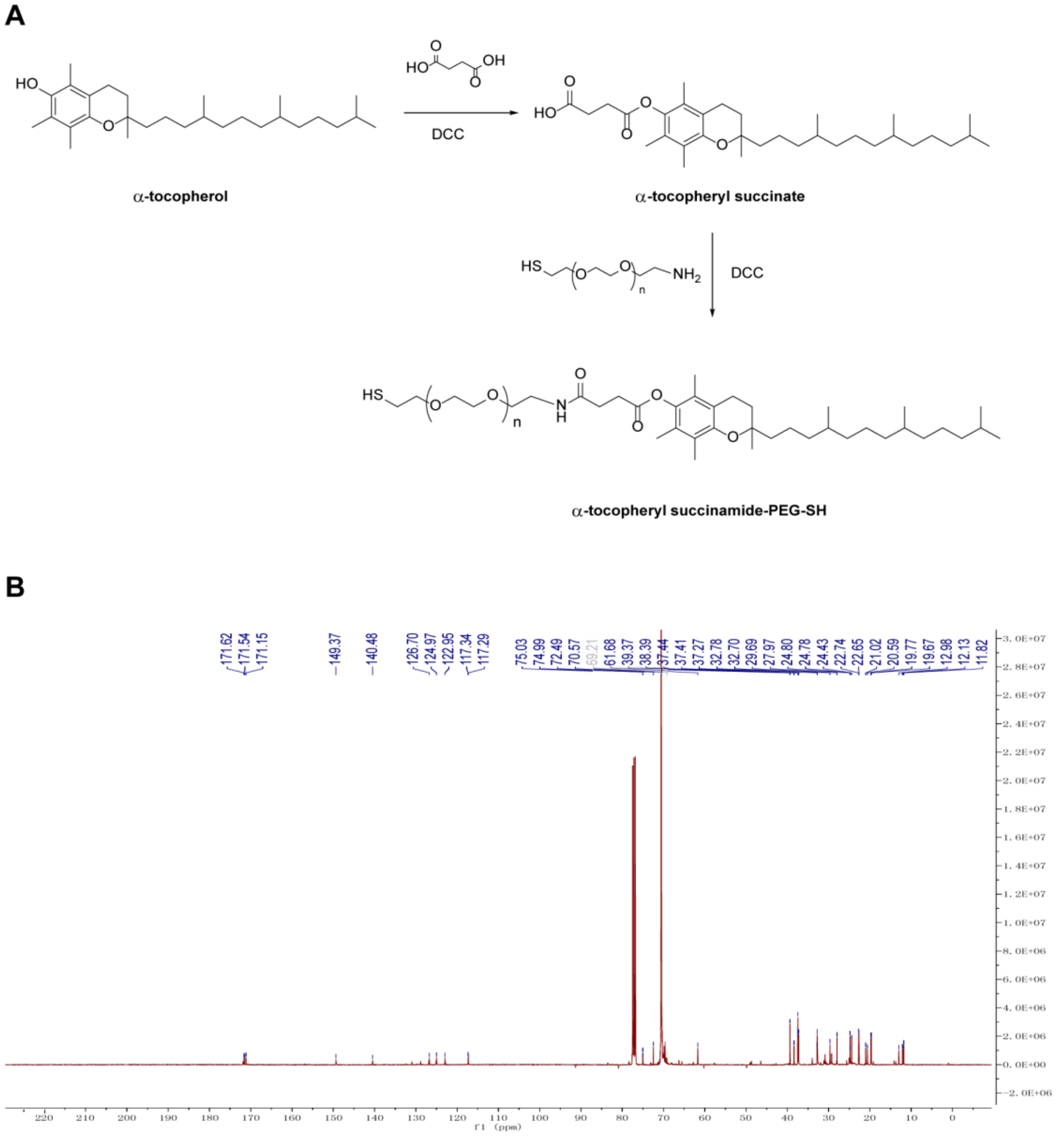

Supplementary Figure 1. Preparation and Characterization of TPGTSA. A. The preparation route of TGPTSA. **B.** ^{13}C -NMR spectrometry of TPGTSA.

The results of Fig. 1 and Supplementary Fig.1 indicated that TPGTSA was successfully synthesized. Intense peaks of ^{1}H-nuclear magnetic resonance (^{1}H-NMR, Fig. 1A, left) for the –NHCO- group (6.55 ppm), the methyl group (2.09, 2.02, 1.98, 0.8 ppm) of α-tocopherol, the methylene group (3.66 ppm) of PEG and (2.96 ppm and 2.90 ppm) of succinate, and the signals of ^{13}C-NMR spectrum (Supplementary Fig. 1B) for the –NHCO- group (171.6 ppm), the phenyl

group, the –COOC-group (171.2 ppm), the methyl group and the methylene group of tocopheryl succinate and the methylene group (70.6 ppm) of PEG indicated that TPGTSA was successfully synthesized. In Fourier transform infrared spectrum (Fig. 1A, middle), the replacement of stretching vibration 1753.3 cm^{-1} of –COOH with 1625.2 cm^{-1} indicated the formation of the -NHCO- bond. In Matrix-assisted laser desorption/ionization-time of flight (MALDI-TOF) mass Spectrometry (Fig. 1A, right), the molecular weight of TPGTSA (2544 Da) was about 512 Da higher than that of NH2-(PEG)n-SH (2032 Da), indicating that the NH2-$(PEG)_n$-SH has been linked with D-α-tocopheryl succinate (530 Da).

Compound characterization data: 1H NMR (500 MHz, Chloroform-d) δ 6.66 – 6.47 (m, 1H), 3.66 (s, 274H), 2.97 (q, J = 8.1, 7.5 Hz, 2H), 2.90 (t, J = 6.6 Hz, 1H), 2.59 (t, J = 6.2 Hz, 5H), 2.09 (s, 3H), 2.02 (d, J = 5.1 Hz, 3H), 1.99 (d, J = 5.2 Hz, 3H), 1.91 – 1.69 (m, 4H), 1.67 – 0.99 (m, 32H), 0.99 – 0.72 (m, 13H). 13C NMR (100 MHz, Chloroform-d) δ 171.6 , 171.2 , 149.4 , 140.5 , 126.7 , 125.0 , 123.0 , 117.3, 75.0 , 72.5 , 70.6 , 69.8 , 61.7 , 39.4 , 38.4, 37.4 , 37.4 , 37.3 , 32.8 , 32.7 , 29.7 , 28.0 , 24.8 , 24.8 , 24.4 , 22.7 , 22.6 , 21.0 , 20.6 , 19.8 , 19.7 , 13.0, 12.1, 11.8. IR: 2886.3 cm-1, 1728.5 cm-1, 1625.2 cm-1, 1467.1 cm-1, 1344.1 cm-1, 1280.9 cm-1, 1148.5 cm-1, 1113.5 cm-1, 963.8 cm-1, 842.5 cm-1, 745.4 cm-1. MALDI-TOF MS (-): 2544.6 m/z.

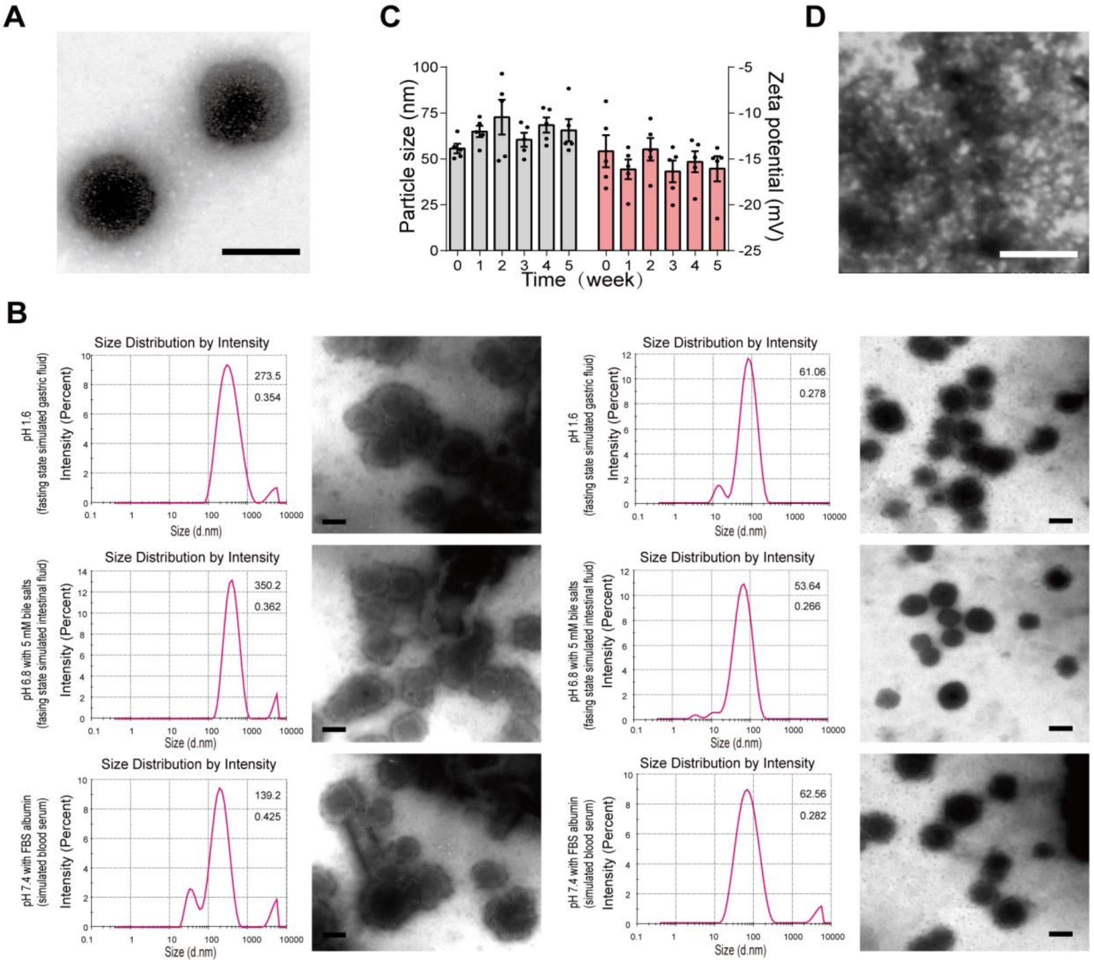

Supplementary Figure 2. Characteristics of BBR-CTA-Mic. A. The representative TEM image of BBR-CTA-Mic. **B.** The representative TEM image and properties (PS, PDI) of BBR-CTA-Mic and BBR-TPGS-Mic in simulated biological fluids of pH 1.6, pH 6.8 with 5mM of bile salts and pH 7.4 with 20% FBS albumin. **C.** PS and ZP of BBR-CTA-Mic in refrigerated conditions (4˚C) for five weeks Data are presented as mean ± SEM (n=5). **D.** The representative TEM image of BBR-CTA-Mic after incubated with 20% hepatic homogenate. Scale bars, 50 nm (A, B, D). Differences between groups were analyzed using unpaired student's t test, two-sided.

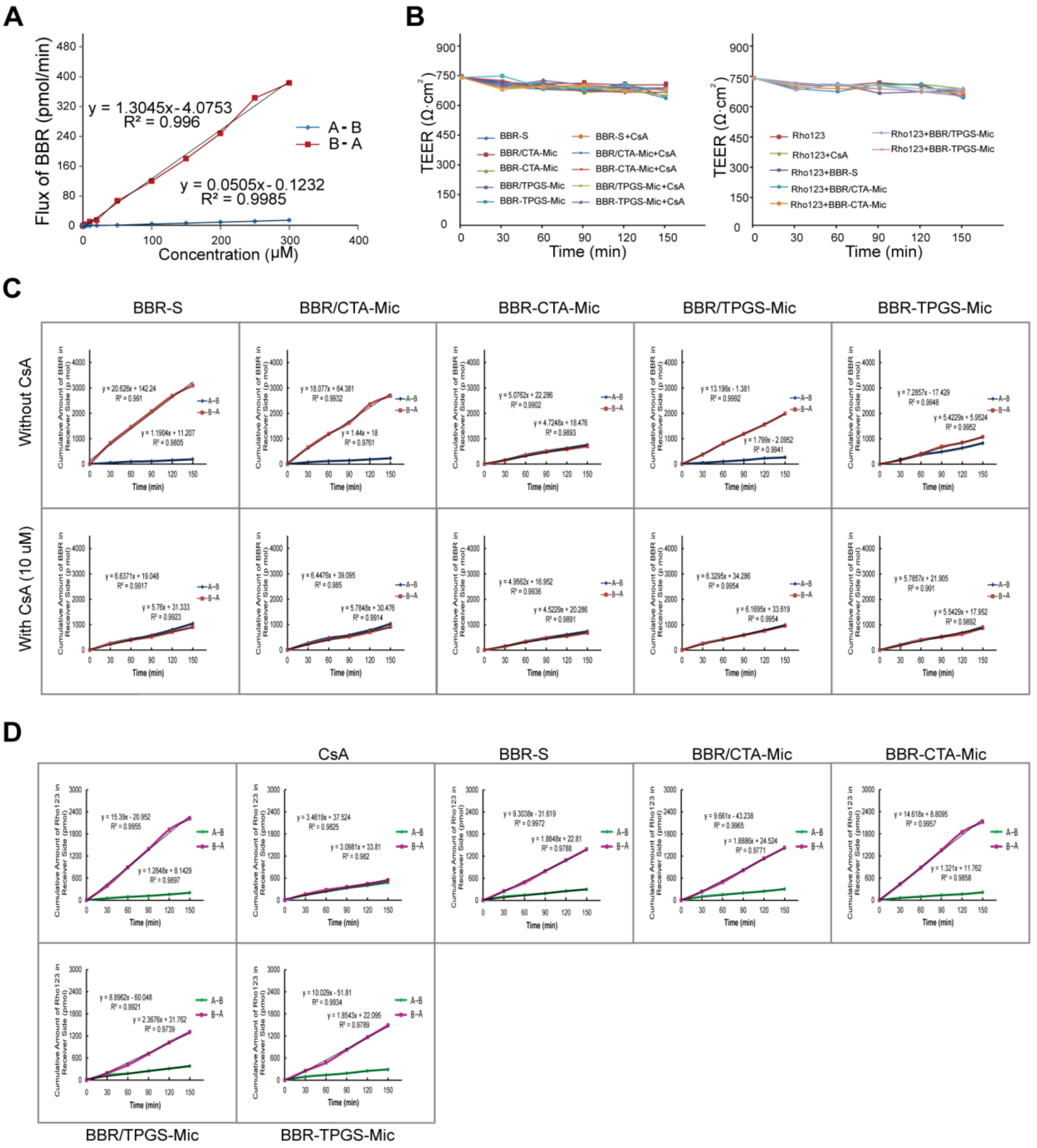

Supplementary Figure 3. Trans-epithelial transport study. A. The apical-to-basolateral (A-B) and basolateral-to-apical (B-A) flux of BBR across Caco-2 cell monolayers were tested as a function of BBR concentration. **B.** TEER values. **C.** A-B and B-A transport of BBR in BBR containing formulations were evaluated with present or absent of CsA (10 μM). **D**. A-B and B-A transport of Rho123 were investigated in the condition of CsA or various BBR containing formulations.

The amounts of BBR transported across the Caco-2 cell monolayers in the apical to

basolateral direction (A-B) and in the basolateral to apical direction (B-A) directions were proportional to a time of 150 min for all tested groups. The transport of BBR in both directions increased linearly with an increasing concentration of BBR over the range of 2-300 μM (Supplementary Fig. 3A), indicating the K_m of BBR in the Caco-2 cells is over 300 μM. To avoid possible cytotoxicity, a concentration of 20 μM was used in the following experiments. The monolayers with TEER higher than 600 $\Omega\cdot cm^2$ were used in the transport experiments (Supplementary Fig. 3B).

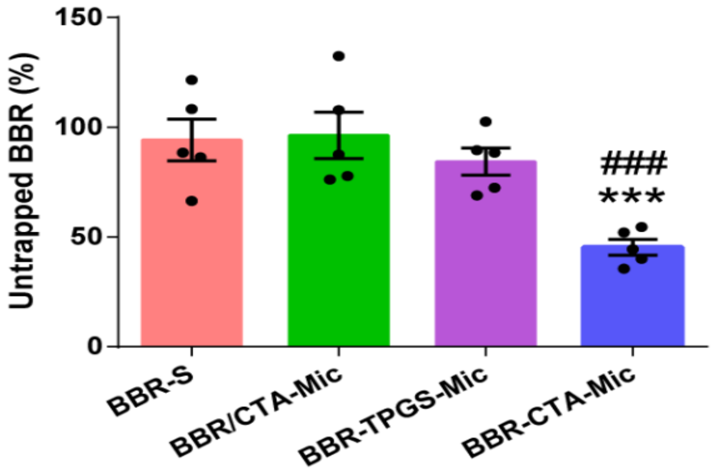

Supplementary Figure 4. Integrity assessment of BBR formulations after trans-epithelial transportation. At the end of tans-epithelial transport experiment, 200 μL aliquots at basolateral chamber were collected and transferred to Amicon® Ultra centrifugation filters (Merck Millipore, Billericia, MA, USA, with 3K Da cut-off) and centrifuged at 5,000 × g for 15 min. The free BBR in filtrate was analyzed using LC/MS/MS. Differences between groups were analyzed using unpaired student's t test, two-sided (mean ±SEM, n = 5; ****p*< 0.001 vs BBR-S; ###*p*< 0.001 vs BBR-TPGS-Mic).

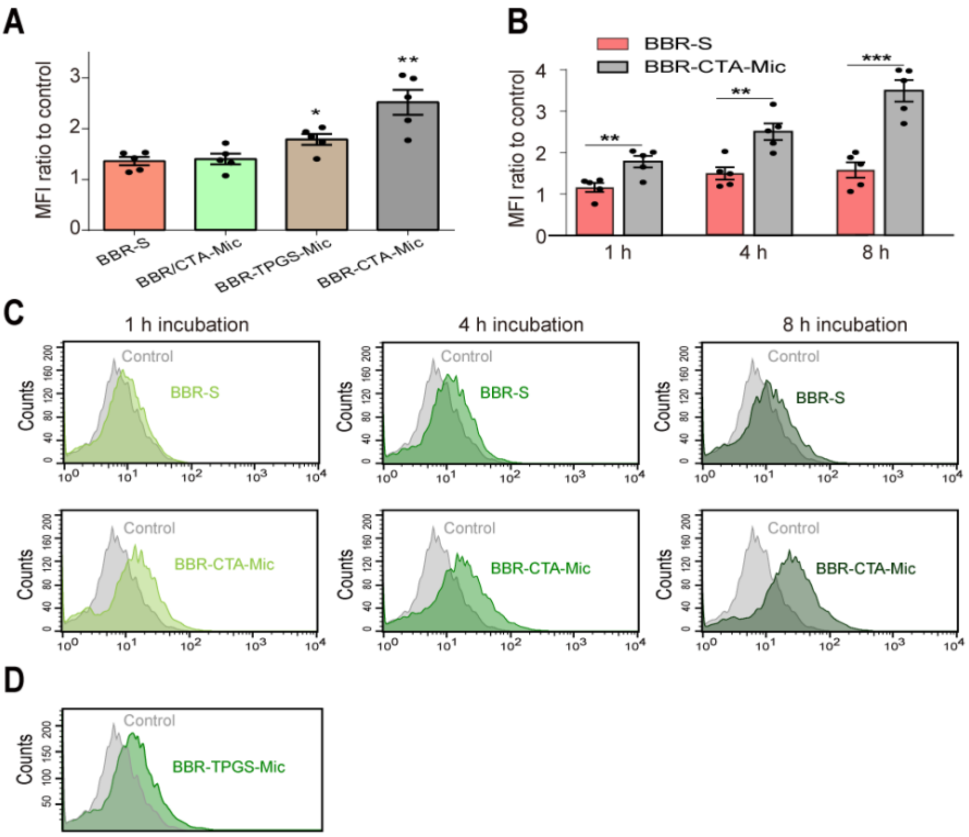

Supplementary Figure 5. Intracellular uptake analysis. The HepG2 cells were treated with various BBR formulations at an equivalent BBR concentration of 1 μg mL^{-1} at 37˚C in 5% CO_2. **A.** Histogram of mean fluorescent intensity (MFI) ratio (vs control) of BBR in HepG2 cells after treated with various BBR formulations for 3 h. (mean±SEM, n = 5; **p* <0.05, ***p* <0.01, vs BBR-S) **B.** The cells were treated with BBR-S or BBR-CTA-Mic for 1, 4 or 8 h, respectively. Histogram and MFI ratio (vs control) of BBR in HepG2 cells obtained from flow cytometry. (mean±SEM, n = 5; ***p* <0.01, ****p*<0.001, vs BBR-S) **C.** Representative flow cytometry diagrams of BBR in HepG2 cells after incubated with BBR-S (up) or BBR-CTA-Mic (down) for 1, 4 or 8 h. **D.** Representative flow cytometry diagram of BBR in HepG2 cells after treated with BBR-TPGS-Mic for 3 h. Differences between groups were analyzed using unpaired student's t test, two-sided.

The cellular uptake of conventional BBR-TPGS-Mic was also tested in hepatocytes. The

result showed that BBR-TPGS-Mic could also increase the uptake of BBR, but not as efficient as the BBR-CTA-Mic did (Supplementary Fig. 5A, 5D). Moreover, the instability of BBR-TPGS-Mic in vivo makes it difficult to arrival liver cell in its intact nanoparticle form.

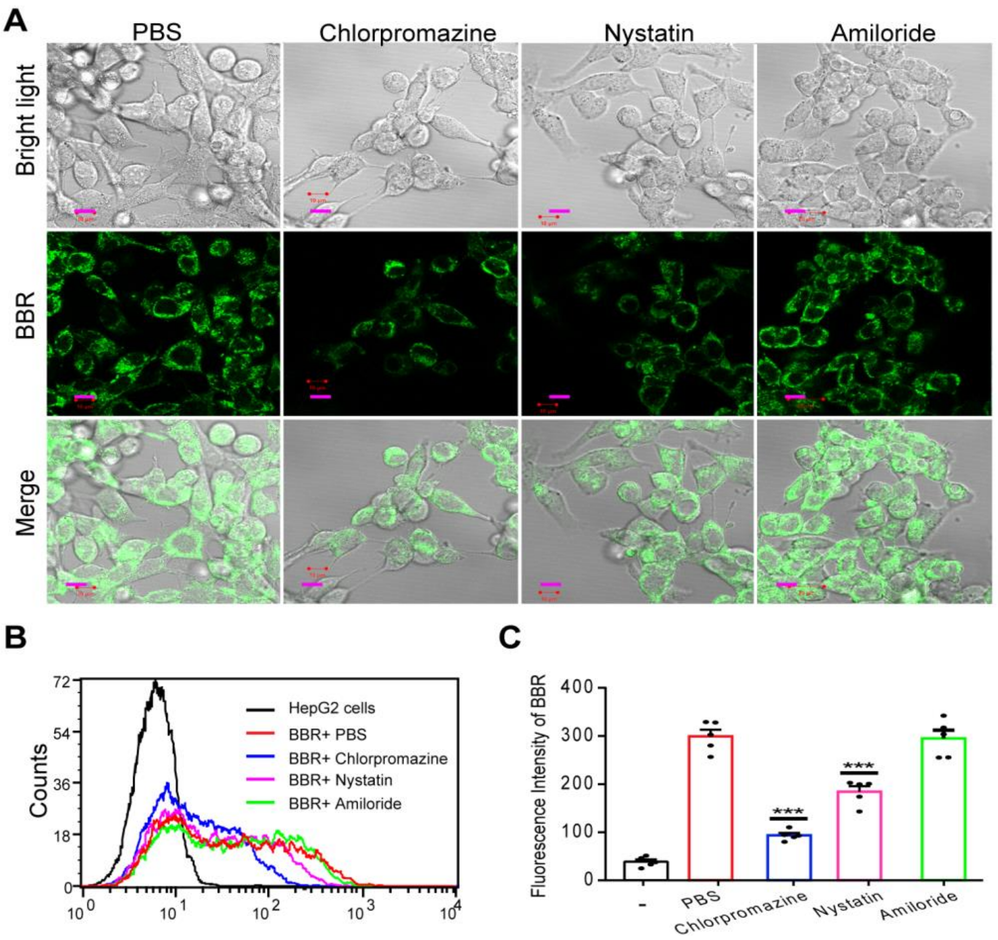

Supplementary Figure 6. Effect of endocytosis inhibitors on the uptake of BBR-loaded CTA-Mics. The HepG2 cells were incubated with BBR-CTA-Mics containing PBS or different endocytosis inhibitors. **A.** Representative fluorescent images of BBR visualized using CLSM. **B.** Representative flow cytometry diagram of BBR in HepG2 cells. **C**. Histogram of mean fluorescent intensity of BBR in HepG2 cells. Data are presented as mean ± SEM (n = 5). **p*< 0.05, ***p*< 0.01, ****p*< 0.001 vs PBS group; Scale bars, 10 μm (A). Differences between groups were analyzed using unpaired student's t test, two-sided.

The mechanism of endocytosis of BBR-CTA-Mic was investigated. The result showed that clathrin-mediated endocytosis was the prominent one for BBR-CAT-Mic uptake, followed by caveolae-mediated endocytosis.

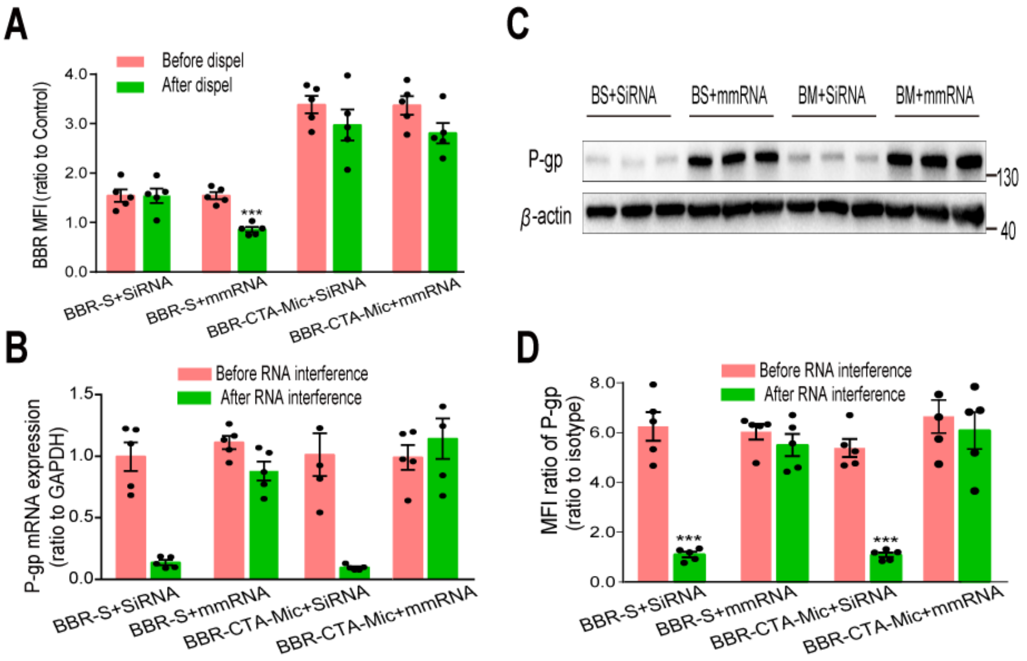

Supplementary Figure 7. P-gp-mediated efflux of BBR-S and BBR-CTA-Mic. The HepG2 cells were pretreated with BBR-S or BBR-CTA-Mic accompanied with P-gp siRNA or mismatch siRNA (mm RNA) (50 nM) for 8 h. Cells were washed with PBS twice and incubated with fresh medium for another 4 h. **A.** Histogram of MFI ratio (vs control) of BBR in HepG2 cells (mean ± SEM, n = 5). **B.** Expression of *P-gp* mRNA was evaluated by RT-PCR. The results were normalized to *GAPDH*. (Mean ± SEM, n = 5) **C.** Expression of P-gp protein was tested using Western blot analysis. The results were normalized to β-actin as density ratio. **D.** Histogram of MFI ratio (vs isotype) of P-gp in HepG2 cells (mean ± SEM, n = 5). *$p < 0.05$, **$p < 0.01$, ***$p < 0.001$. Differences between groups were analyzed using unpaired student's t test, two-sided.

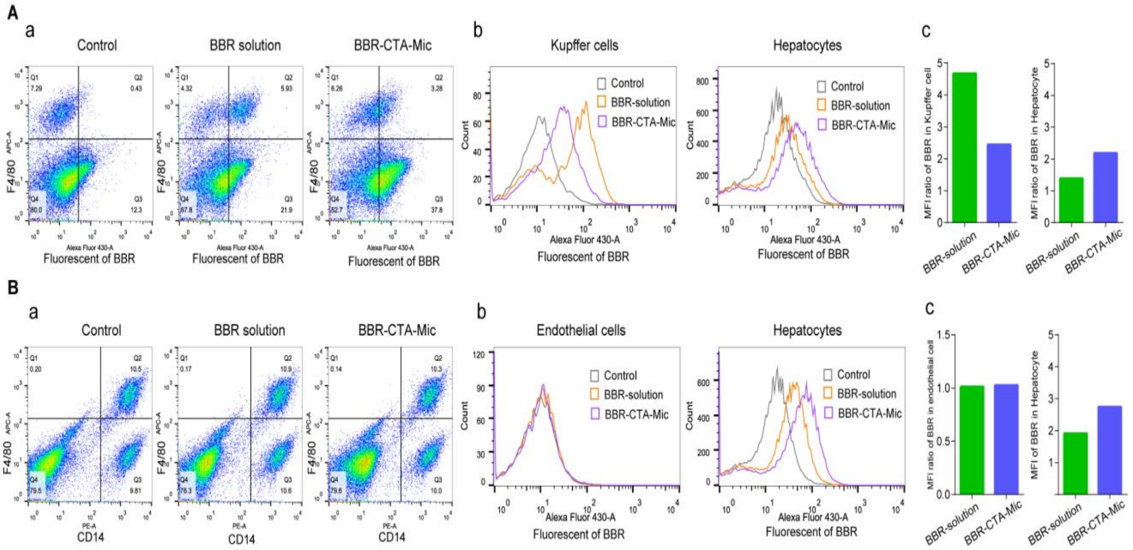

Supplementary Figure 8. Liver cell distribution after BBR-CTA-Mic or BBR-S treatment. A. Drug uptake by kupffer cells and hepatocytes in liver tissue. **A-a:** Representative plots of Kupffer cells ($F4/80^+$, up) and non-kupffer cells (most of which are hepatocytes, bottom), X-axis represents fluorescence of BBR. **A-b:** Histogram of BBR uptake by kupffer and hepatocytes; **A-c:** Mean fluorescent index ratio (MFI ratio vs control) of BBR in kupffer cells and hepatocyte. **B.** Drug uptake by liver sinusoidal endothelial cells (LSECs) and hepatocytes in liver tissue. **B-a:** Representative plots of LSECs ($CD14^+F4/80^-$, bottom right) and hepatocyte (bottom left). **B-b:** Histogram of BBR uptake by LSECs and hepatocytes; **B-c:** Mean fluorescent index ratio (vs control) of BBR in LSECs and hepatocyte.

C57BL/6J mice were treated with BBR-CTA-Mic (50mg kg^{-1} of BBR) or BBR-solution (50 mg kg^{-1} of BBR) by gavage, mice treated with PBS were used as control. Four hours after administration, the mice were anesthetized by an intraperitoneal injection of 30 mg kg^{-1} pentobarbital. The liver tissues were harvested and liver cell suspension was obtained according to protocol[1-4]. Then, the liver cell suspensions were stained with APC-conjugated rat F4/80 antibody (1:50, ab105155, Abcam, USA; or isotype control antibody: 1:50, 553988, BD, USA) for 30 min

in order to evaluate BBR uptake in Kupffer cells and non-Kupffer cells (most of which are hepatocytes), or stained simultaneously with APC-conjugated rat F4/80 antibody and PE-conjugated rat CD14 (1:40, 12-0141-82, Thermofisher, USA) to analyze the uptake of BBR in other liver nonparenchymal cells, most of which are liver sinusoidal endothelial cells. The BBR uptake in different cell lines was detected using flow cytometry.

The results showed that the BBR content in the Kupffer cells of the mice treated with BBR-CTA-Mic was less than that from the mice treated with BBR solution (Supplementary Fig. 8A), indicating a reduced uptake / elimination of BBR by the kupffer cells in liver. Furthermore, other liver nonparenchymal cells (mainly the liver sinusoidal endothelial cells, LSECs) showed almost no uptake of BBR, in BBR solution treated or BBR-CTA-Mic-treated mice (Supplementary Fig. 8B). It is noteworthy that, in the liver of the BBR-CTA-Mic treated mice, the average BBR content in hepatocyte population was similar to that of Kupffer cells (Supplementary Fig. 8A-c). As the number of hepatocytes in liver is many times more than that of the other types of cells, the sum of BBR in total hepatocytes (mediated through the CTA-Mic entrapment) should be much more than that in other types of cells. Regarding the possible mechanism, these results might be interpreted by: 1) The chemical and physical feature of the BBR-CTA-Mic (such as PEG chains on the surface of the carrier, as well as the diameter of the particles is within 20-100 nm arrange) could help it to evade the elimination by reticular-endothelial system in liver (such as the Kupffer's cells)[5-12]; 2) The increased penetration and accumulation of BBR in hepatocytes was facilitated by CTA-Mics. CD14 was used for LSECs staining in the experiments, as CD14 was reported to be a surface marker for the LSECs[13-17]. However, as kupffer cells are also positive for CD14, double-staining of the homogenized mice liver cell suspension (mainly containing

hepatocytes, kupffer cells and LSECs) was done with CD14 antibody as well as F4/80 antibody (for kupffer cell specific marker, identical to that used in the Supplementary Fig. 8A), in which the $CD14^{+}F480^{-}$ cells could be considered as LSECs and the $CD14^{+}F4/80^{+}$ cells as kupffer cells. Thus, we could easily identify LSECs in the cell suspension ($CD14^{+}F480^{-}$ cells at the IV quadrant, the lower-right corner, see Supplementary Fig. 8B-a). Then, we analyzed the BBR level in the LSECs after gating the $CD14^{+}F480^{-}$ cells. As shown in the Supplementary Fig. 8B-b and 8B-c, the $CD14^{+}F4/80^{-}$ cells showed almost no uptake of BBR, in BBR solution or BBR-CTA-Mic treated mice. CD146 might be a better marker for the LSECs, but double staining analysis is also needed as some lymphocytes are positive for CD146 staining[18-20]. We are planning to conduct the experiment, aiming for detailed cell category recognition in BBR-CTA-Mic uptake.

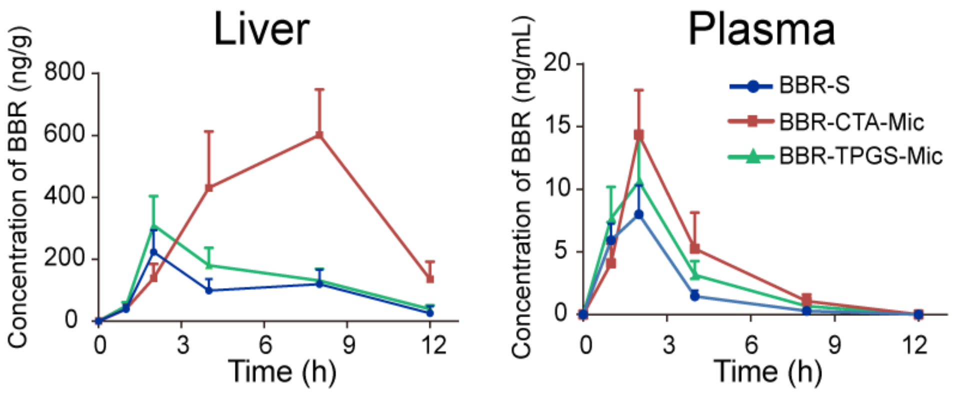

Supplementary Figure 9. BBR content in plasma and liver tissue. C57BL/6J mice were treated with BBR-S, BBR-CTA-Mic or BBR-TPGS-Mic (50 mg kg^{-1} of BBR) by gavage. BBR content in plasma and liver tissues at different time points after drug administration was evaluated using LC-MS/MS. Data are presented as mean ± SEM (n = 5).

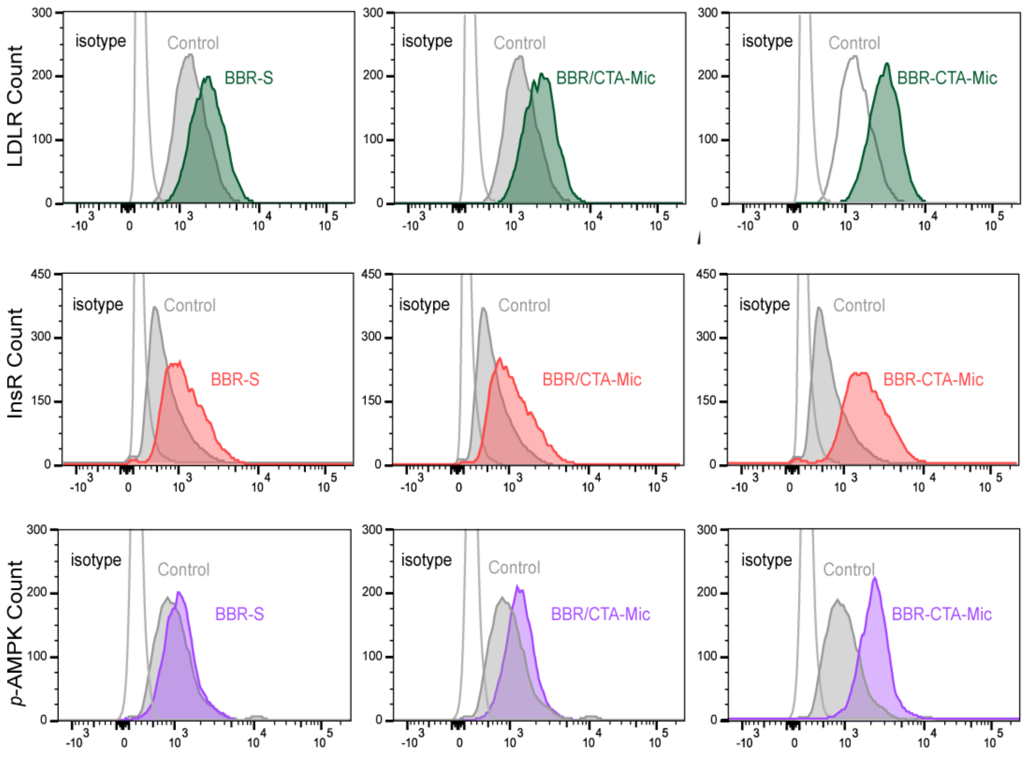

Supplementary Figure 10. In vitro pharmacological effect. Representative flow cytometry diagraph of p-AMPK, InsR and LDLR protein in HepG2 cells treated with different BBR formulations (1 μg ml^{-1} of BBR) for 8 h.

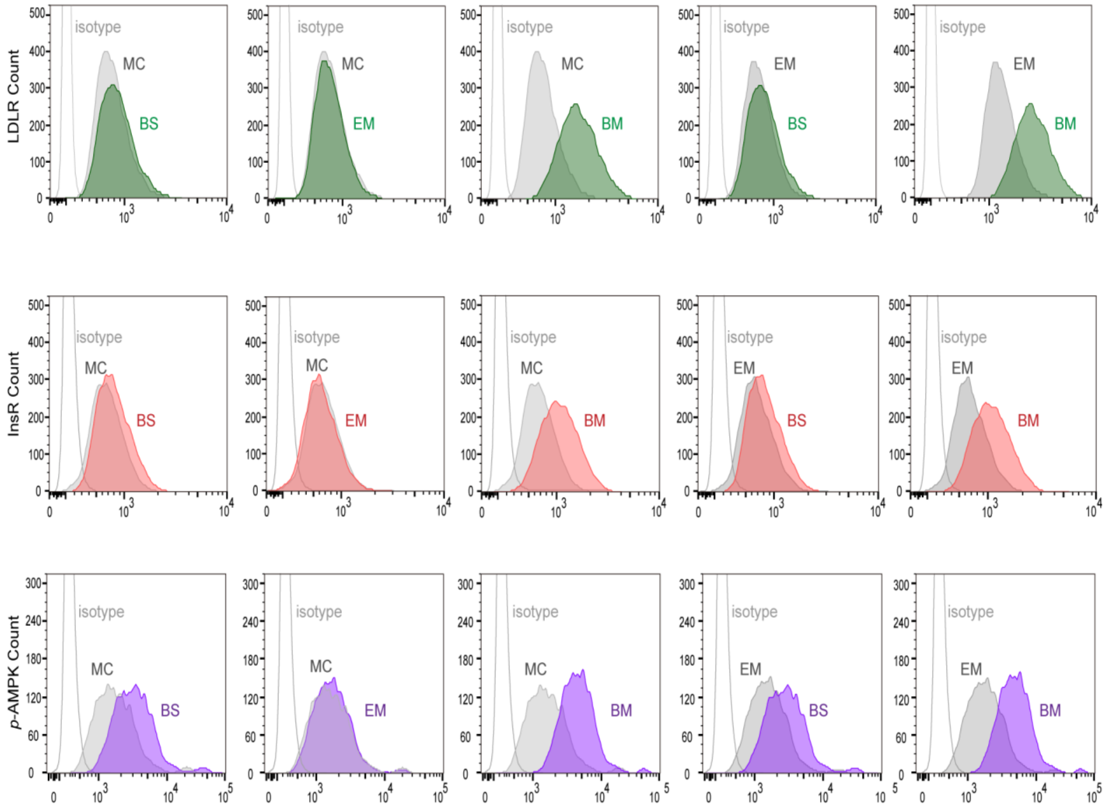

Supplementary Figure 11. In vivo gene stimulation effect analysis. Representative flow cytometry diagraph of p-AMPK, InsR and LDLR protein in liver tissue of HFD-fed C57BL/6J mice treated with different BBR formulations (50 mg kg^{-1} day^{-1} of BBR) by gavage for 2 months.

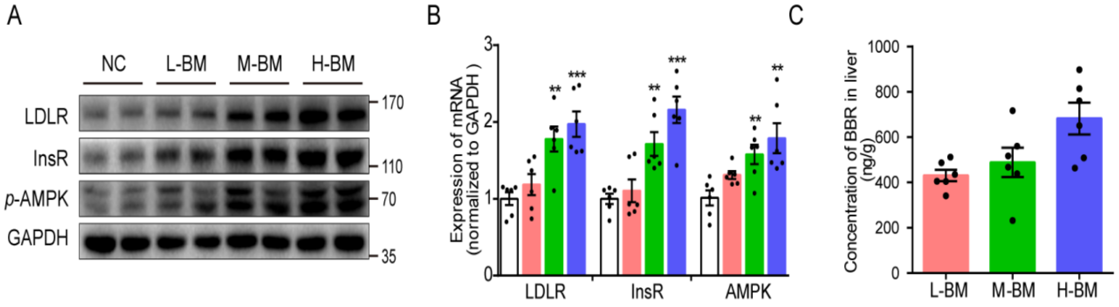

Supplementary Figure 12. Effect of BBR-CTA-Mic in BBR dose response. BBR-CTA-Mic with gradient amount of BBR (low dose 25 mg (L-BM), middle dose 50 mg (M-BM) and high dose 75 mg (H-BM) kg^{-1} day^{-1} of BBR) were administered to C57BL/6J (6 weeks; 18-20 g) for two weeks by gavage, untreated mice (NC group) was used as control. 4h after the last dose, the mice were anesthetized and the liver tissues were harvested. The expression of LDLR, InsR and AMPK were tested using Western blot and RT-PCR. The BBR content in liver was also analyzed by LC-MS/MS. **A.** The protein expression of p-AMPK, InsR and LDLR was tested using Western blot analysis. The results were normalized to GAPDH. **B.** The mRNA expression of *AMPK*, *InsR* and *LDLR* was evaluated by RT-PCR. The results were normalized to *GAPDH*. **C.** The liver BBR content analyzed using LC-MS/MS. Data are presented as mean ± SEM (n = 6). Differences between groups were analyzed using unpaired student's t test, two-sided. (*$p<$ 0.05, **$p<$ 0.01, ***$p<$ 0.001, vs mice in NC)

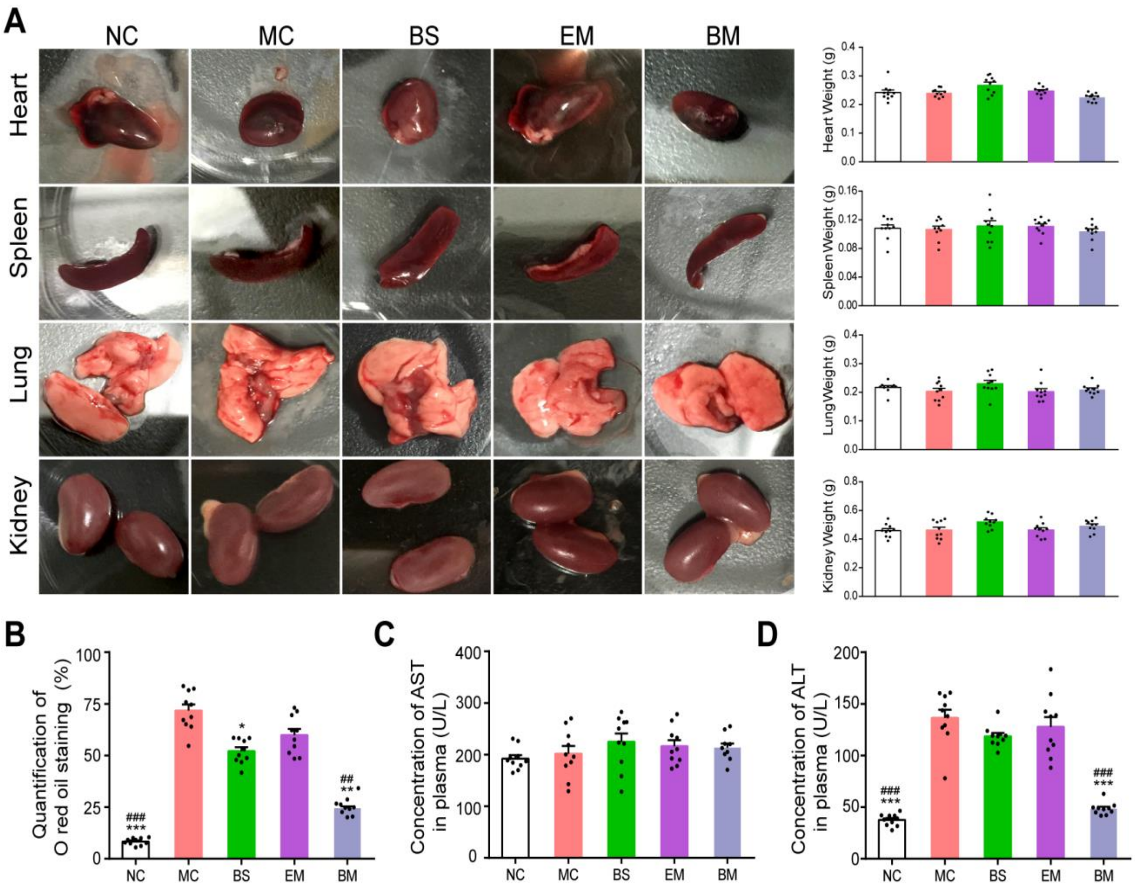

Supplementary Figure 13. In vivo pharmacodynamics analysis. C57BL/6J mice were treated with various BBR formulations (50 mg kg^{-1} day^{-1} of BBR) by gavage. **A.** Representative pictures and weight change of heart, spleen, lung and kidney. **B.** Qualification of oil red O staining. **C.** Concentrations of AST in plasma of mice. **D.** Concentrations of ALT in plasma of mice. Data are presented as mean ± SEM (n = 10). Differences between groups were analyzed using unpaired student's t test, two-sided. (*p< 0.05, **p< 0.01, ***p< 0.001, vs mice in MC group; $^{\#}p$ <0.05, $^{\#\#}p$ <0.01, $^{\#\#\#}p$ <0.001, vs mice in EM group)

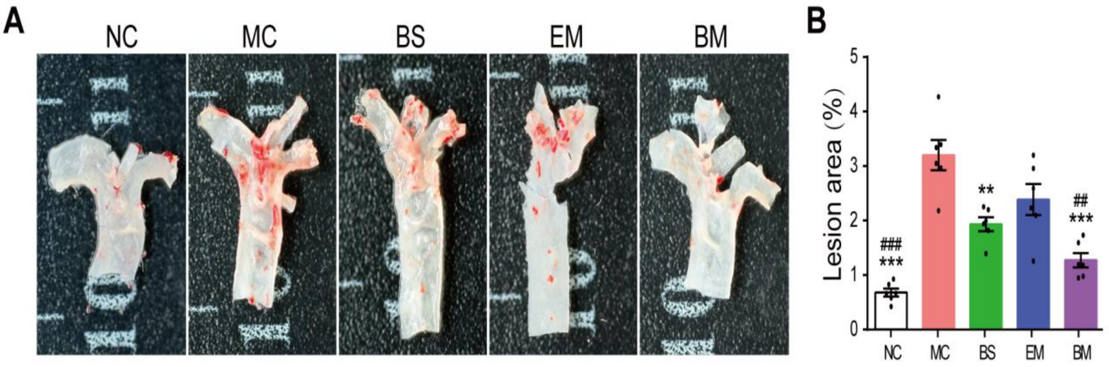

Supplementary Figure 14. Atherosclerotic lesion assessment. HFD-fed C57BL/6J mice treated with various BBR formulations (50 mg kg^{-1} day^{-1} of BBR) by gavage. Aortas samples were perfused with 0.9% NaCl solution and dissected stained with Oil Red O. **A.** Representative images of aortic arch lesion in C57BL/6J mice treated with different BBR formulations. **B.** Aortic arch atherosclerotic lesions were measured as percentage of luminal diameter. Data are presented as mean ± SEM (n = 6). Differences between groups were analyzed using unpaired student's t test, two-sided. (*$p< 0.05$, **$p< 0.01$, ***$p< 0.001$, vs mice in MC group; $^{\#}p$ <0.05, $^{\#\#}p$ <0.01, $^{\#\#\#}p$ <0.001, vs mice in EM group)

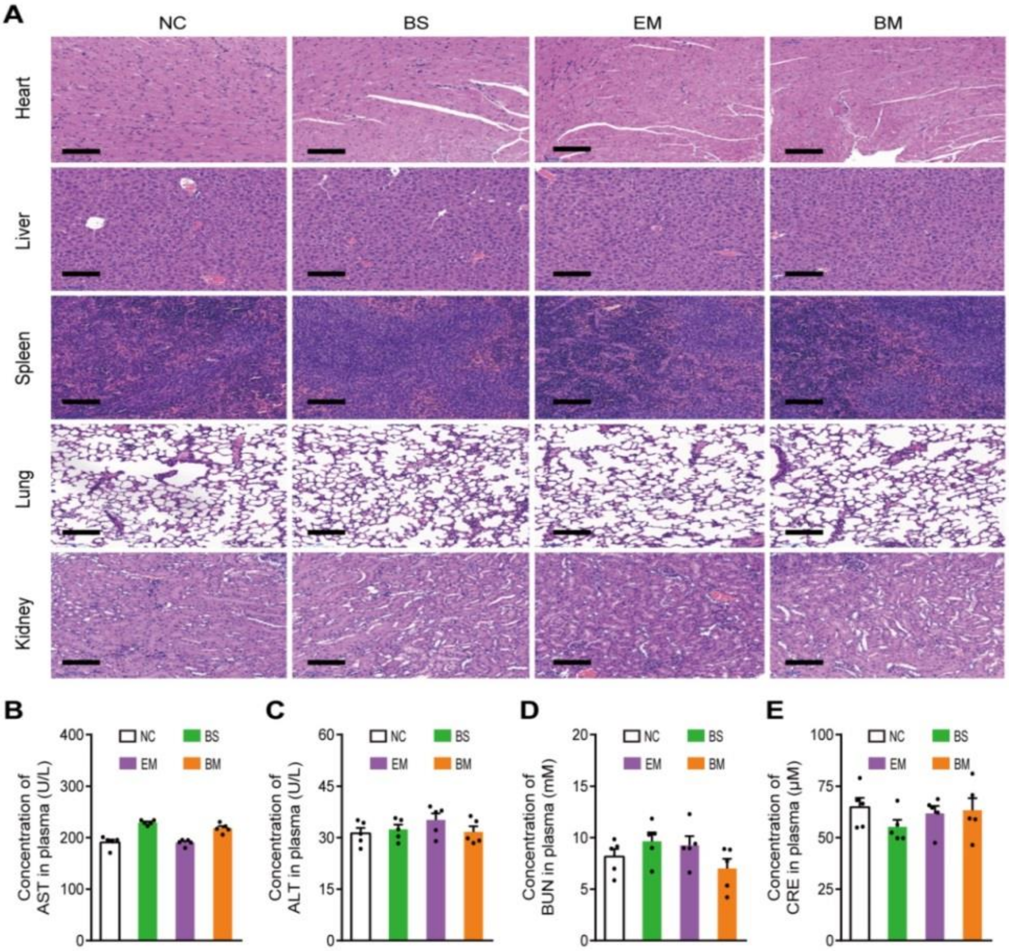

Supplementary Figure 15. Long-term safety analysis. C57BL/6J mice received various BBR formulations (50 mg kg^{-1} day^{-1} of BBR) for 4 months by gavage. **A.** HE staining of main tissues. (Scale bars, 100 μm) **B.** Concentration of AST. **C.** Concentrations of ALT. **D.** Concentrations of BUN. **E.** Concentrations of CRE. Data are presented as mean ± SEM (n = 5). Scale bars: 100 μm (A). Differences between groups were analyzed using unpaired student's t test, two-sided.

The results of long-term safety analysis showed that there were no obvious histological differences of major organs between the treated and untreated mice. No significant differences were detected between medication group and the untreated control group in the plasma levels of ALT, AST, creatinine (CRE) and blood urea nitrogen (BUN), indicating a good tissue compatibility of the delivery system designed in this study.

Supplementary Tables

Supplementary Table 1. Trans-epithelial transport study

	inhibitors	Flux speed (A-B) (pmol min^{-1})	Flux speed (B-A) (pmol min^{-1})	Papp (A-B) ($\times 10^{-6}$ cm s^{-1})	Papp (B -A) ($\times 10^{-6}$ cm s^{-1})	ER
BBR-S	Control	1.18±0.10	20.62±1.60	0.87±0.04	15.06±0.86	17.31
	CsA	6.66±0.22	5.77±0.17	4.94±0.15	4.25±0.22	0.87
BBR/CTA-Mic	Control	1.40±0.11	18.12±0.97	1.05±0.05	13.59±0.59	12.56
	CsA	6.46±0.39	5.64±0.18	4.70±0.10	4.32±0.11	0.90
BBR-CTA-Mic	Control	5.12±0.25	4.68±0.07	3.63±0.23	3.47±0.10	0.93
	CsA	4.90±0.11	4.51±0.15	3.61±0.13	3.33±0.11	0.91
BBR/TPGS-Mic	Control	1.82±0.09	13.20±0.17	1.31±0.06	9.82±0.37	7.33
	CsA	6.34±0.19	6.21±0.19	4.65±0.08	4.52±0.11	0.97
BBR-TPGS-Mic	Control	7.23±0.20	5.40±0.15	4.02±0.15	5.40±0.13	1.35
	CsA	5.79±0.09	5.59±0.12	4.26±0.16	4.02±0.07	0.96
Rho123	Control	1.30±0.07	15.37±0.62	1.94±0.16	23.05±1.04	12.02
	CsA	3.10±0.08	3.43±0.10	4.63±0.19	5.09±0.09	1.12
	BBR-S	1.89±0.03	9.29±0.21	2.76±0.04	13.85±0.62	4.92
	BBR/CTA-Mic	1.88±0.05	9.56±0.73	2.86±0.39	14.26±1.11	5.11
	BBR-CTA-Mic	1.31±0.06	14.25±0.88	1.99±0.13	22.61±1.62	11.08
	BBR/TPGS-Mic	2.37±0.10	8.75±0.28	3.51±0.10	13.03±0.93	3.76
	BBR-TPGS-Mic	1.86±0.02	10.41±0.54	2.72±0.04	14.92±0.98	5.41

Supplementary Table 2. Primer sequences of genes in HepG2 cells and C57BL/J mice

Species	Gene	Forward primer	Reversed primer
Human	*LDLR*	CCACTCGCCCAAGTTTACCT	TGGTTGTGTGCTGTGTCCTT
	InsR	GACAACGAGGAGTGTGGAGA	TACAGATGGTCGGGCAAACT
	AMPK	TGGAGCAGTGGGGTTATTCT	GATTGTGGCCCTCTTCATGG
	AKT	AGAAGCAGGAGGAGGAGGAG	CTTGGCCACGATGACTTCCT
	P-gp	ACAGAAAGCGAAGCAGTGGT	ATGGTGGTCCGACCTTTTC
	TNF-α	CCTCTTCTCCTTCCTGATCGT	TGCTACAACATGGGCTACAG
	IL-6	GACCCAACCACAAATGCCAG	GGAACTCCTTAAAGCTGCGC
	GAPDH	GGTGAAGGTCGGAGTCAACG	TGGGTGGAATCATATTGGAACA
Mouse	*LDLR*	AAGAATGTGGTGGCTCTCGA	TGTCCAGTAGATGTTGCGGT
	InsR	ATCACTCAGGGCAAGCTCTT	CCCAGGAGATCTCGGAAGTC
	AMPK	ACCTGACTCTTTCCTGGACG	AATGCCATTTTGCCTTCCGT
	AKT	GCAAGGAGGGGATCAAGGAC	CGGCCACACATCATCTCGTA
	TNF-α	CACAGAAAGCATGATCCGCG	ACTGATGAGAGGGAGGCCAT
	IL-6	AGCCAGAGTCCTTCAGAGAGA	GGATGGTCTTGGTCCTTAGCC
	GAPDH	CTCCCACTCTTCCACCTTCG	TAGGGCCTCTCTTGCTCAGT

Supplementary References

1. Kegel, V., Deharde, D., Pfeiffer, E., Zeilinger, K., Seehofer, D. & Damm, G. Protocol for Isolation of Primary Human Hepatocytes and Corresponding Major Populations of Non-parenchymal Liver Cells. *J Vis. Exp.* e53069 (2016).
2. Cabral, F. et al. Purification of Hepatocytes and Sinusoidal Endothelial Cells from Mouse Liver Perfusion. *J Vis. Exp.* (2018).
3. Edstrom, S., Ekman, L., Ternell, M. & Lundholm, K. Isolation of mouse liver cells: perfusion technique and metabolic evaluation. *Eur. Surg. Res.* **15**, 97-102 (1983).
4. Aparicio-Vergara, M., Tencerova, M., Morgantini, C., Barreby, E. & Aouadi, M. Isolation of Kupffer Cells and Hepatocytes from a Single Mouse Liver. *Methods. Mol. Biol.* **1639**, 161-171 (2017).
5. Pasut, G. et al. Polyethylene glycol (PEG)-dendron phospholipids as innovative constructs for the preparation of super stealth liposomes for anticancer therapy. *J Control Release* **199**, 106-113 (2015).
6. Harris, J. M. & Chess, R. B. Effect of pegylation on pharmaceuticals. *Nat. Rev. Drug. Discov.* **2**, 214-221 (2003).
7. Wang, Y. et al. Novel galactosylated biodegradable nanoparticles for hepatocyte delivery of oridonin. *Int. J Pharm.* **502**, 47-60 (2016).
8. Xie, J., Xu, C., Kohler, N., Hou, Y. & Sun, S. Controlled PEGylation of Monodisperse Fe3O4 Nanoparticles for Reduced Non-Specific Uptake by Macrophage Cells. *Advanced Materials* **19**, 3163-3166 (2007).
9. Yang, A. S., Liu, W. & Yang, X. L. Serum Proteins Opsonization and Phagocytic Uptake of PEG-Modified PLGA Nanoparticles: Effect of Particle Size, *Adv. Mat. Res.* Vols. **393-395**, 939-942 (2012).
10. Yu, S. S. et al. Size- and charge-dependent non-specific uptake of PEGylated nanoparticles by macrophages. *Int. J Nanomedicine* **7**, 799-813 (2012).
11. Bazile, D., Prud'homme, C., Bassoullet, M. T., Marlard, M., Spenlehauer, G., Veillard, M. & Stealth, M. PEG-PLA nanoparticles avoid uptake by the mononuclear phagocytes system. *J Pharm. Sci* . **84**, 493-498 (1995).
12. Bae, Y. & Kataoka, K. Intelligent polymeric micelles from functional poly(ethylene glycol)-poly(amino acid) block copolymers. *Adv. Drug. Deliv. Rev.* **61**, 768-784 (2009).
13. Jersmann, H. P., Hii, C. S., Hodge, G.L. & Ferrante, A. Synthesis and surface expression of CD14 by human endothelial cells. *Infect. Immun.* **69**, 479-485 (2001).
14. Scoazec, J. Y. & Feldmann, G. In situ immunophenotyping study of endothelial cells of the human hepatic sinusoid: results and functional implications. *Hepatology* **14**, 789-797 (1991).
15. Xu, B. et al. Capillarization of hepatic sinusoid by liver endothelial cell-reactive autoantibodies in patients with cirrhosis and chronic hepatitis. *Am. J Pathol.* **163**, 1275-1289 (2003).

16 Wang, Z., Wang, L., Xie, L. F., Fan, J. Y. & Xu, C. S. Isolation, purification and identification of sinusoidal endothelial cells in rat liver. *Henan Science* **27** 930-934 (2009).

17 liu, B., Fu, T. S., Tang, L. & He, F. C. An improved method for isolation, cultivation and identification of liver sinusoidal endothelial cells in mouse. *Chinese J cell biol.* 3, 437-442 (2009).

18. Elshal, M. F., Khan, S. S., Takahashi, Y., Solomon, M. A. & McCoy, J. P. CD146 (Mel-CAM), an adhesion marker of endothelial cells, is a novel marker of lymphocyte subset activation in normal peripheral blood. *Blood* **106**, 2923-2924 (2005).

19. Elshal, M. F. et al. A unique population of effector memory lymphocytes identified by CD146 having a distinct immunophenotypic and genomic profile. *BMC Immunol.* **8**, 29 (2007).

20. Covas, D. T. et al. Multipotent mesenchymal stromal cells obtained from diverse human tissues share functional properties and gene-expression profile with CD146+ perivascular cells and fibroblasts. *Exp. Hematol*. **36**, 642-654 (2008).
