# Supplementary material for: Liver-target nanotechnology facilitates berberine to ameliorate cardio-metabolic diseases
Source: Nat Commun. 2019 Apr 30;10:1981. doi: 10.1038/s41467-019-09852-0 (PMC6491597; doi:10.1038/s41467-019-09852-0)
Supplement: Supplementary file 6 — Reporting Summary [file 41467_2019_9852_MOESM6_ESM.pdf]

nature research

Corresponding author(s): Lu-Lu Wang, Wen-Sheng Zheng, Yan-Xing Han and Jian-Dong Jiang

Last updated by author(s): Mar 7, 2019

Reporting Summary

Nature Research wishes to improve the reproducibility of the work that we publish. This form provides structure for consistency and transparency in reporting. For further information on Nature Research policies, see Authors & Referees and the Editorial Policy Checklist.

Statistics

For all statistical analyses, confirm that the following items are present in the figure legend, table legend, main text, or Methods section.

n/a	Confirmed	
☐	☒	The exact sample size (*n*) for each experimental group/condition, given as a discrete number and unit of measurement
☐	☒	A statement on whether measurements were taken from distinct samples or whether the same sample was measured repeatedly
☐	☒	The statistical test(s) used AND whether they are one- or two-sided<br>*Only common tests should be described solely by name; describe more complex techniques in the Methods section.*
☐	☒	A description of all covariates tested
☐	☒	A description of any assumptions or corrections, such as tests of normality and adjustment for multiple comparisons
☐	☒	A full description of the statistical parameters including central tendency (e.g. means) or other basic estimates (e.g. regression coefficient) AND variation (e.g. standard deviation) or associated estimates of uncertainty (e.g. confidence intervals)
☐	☒	For null hypothesis testing, the test statistic (e.g. *F*, *t*, *r*) with confidence intervals, effect sizes, degrees of freedom and *P* value noted<br>*Give P values as exact values whenever suitable.*
☒	☐	For Bayesian analysis, information on the choice of priors and Markov chain Monte Carlo settings
☒	☐	For hierarchical and complex designs, identification of the appropriate level for tests and full reporting of outcomes
☐	☒	Estimates of effect sizes (e.g. Cohen's *d*, Pearson's *r*), indicating how they were calculated

Our web collection on statistics for biologists contains articles on many of the points above.

Software and code

Policy information about availability of computer code

Data collection	Image-Pro-Plus 6.0, FlowJo_V10, Living Image 6.4.2, Image Lab 4.0
Data analysis	GraphPad Prism Software Version 5.0a(GraphPad, San Diego, CA)

For manuscripts utilizing custom algorithms or software that are central to the research but not yet described in published literature, software must be made available to editors/reviewers. We strongly encourage code deposition in a community repository (e.g. GitHub). See the Nature Research guidelines for submitting code & software for further information.

Data

Policy information about availability of data

All manuscripts must include a data availability statement. This statement should provide the following information, where applicable:

- Accession codes, unique identifiers, or web links for publicly available datasets
- A list of figures that have associated raw data
- A description of any restrictions on data availability

All data generated or analyzed during this study are included in this published article (and its supplementary information files). A reporting summary for this article is available as a Supplementary Information file. The source data underlying Figs 1C, 1D, 3B, 4B, 4C, 5B, 5C, 6A, 6B, 8A, 8D, 8E and Supplementary Figs 2B, 4, 5A, 5B, 6C, 7A-D, 9, 12A-C, 13A-D, 14B, 15B-E, Supplementary Table 1 are provided as a Source Data file.

Field-specific reporting

Please select the one below that is the best fit for your research. If you are not sure, read the appropriate sections before making your selection.

☒ Life sciences ☐ Behavioural & social sciences ☐ Ecological, evolutionary & environmental sciences

For a reference copy of the document with all sections, see nature.com/documents/nr-reporting-summary-flat.pdf

Life sciences study design

All studies must disclose on these points even when the disclosure is negative.

Sample size	The sample size in each experiment was chosen to ensure adequate power to detect a pre-specified effect.
Data exclusions	Values detected by the two folds of standard deviation were discarded.
Replication	All measurements were performed in 3~5 times under the same circumstances.
Randomization	Animals were randomly assigned to experimental groups according to their body weights.
Blinding	Studies were not blinded to investigators.

Reporting for specific materials, systems and methods

We require information from authors about some types of materials, experimental systems and methods used in many studies. Here, indicate whether each material, system or method listed is relevant to your study. If you are not sure if a list item applies to your research, read the appropriate section before selecting a response.

Materials & experimental systems

n/a	Involved in the study	
☐	☒	Antibodies
☐	☒	Eukaryotic cell lines
☒	☐	Palaeontology
☐	☒	Animals and other organisms
☒	☐	Human research participants
☒	☐	Clinical data

Methods

n/a	Involved in the study	
☒	☐	ChIP-seq
☐	☒	Flow cytometry
☒	☐	MRI-based neuroimaging

Antibodies

Antibodies used

Antibodies used in Western Blot:
Primary antibodies:LDLR (1:1000, 10785-1-AP), P-gp (1:1000, 22336-1-AP), IL-6 (1:1000, 21865-1-AP), TNF-α (1:1000, 60291-1-Ig), GAPDH (1:1000, 10494-1-AP) (Proteintech, USA); p-AMPK (1:1000, ab133448), AMPK (1:1000, ab80039), p-InsR (1:1000, ab60946) (Abcam, UK); p-AKT (1:2000, #4060), AKT (1:2000, #2920), β-actin (1:1000, #4970) (Cell Signaling, USA) or InsR (1:200, sc-57342, Santa Cruz, USA). Secondary antibody:HRP conjugated secondary antibodies(1:5000, #7076, #7074, Cell Signaling, USA).
Antibodies used in flow cytometry analysis:
PE-conjugated mouse anti-human P-gp antibody (1:50, ab93590, Abcam, UK) or isotype control antibody (1:50, #61656, Cell Signaling, USA),Alexa Fluor 488 conjugated rabbit LDLR monoclonal antibody (1:500, ab196377, Abcam, UK) or isotype control antibody (1:500, ab199091, Abcam, UK),rabbit p-AMPK monoclonal antibody (1:50, ab23875, Abcam, UK) or isotype control (1:50, ab172730, Abcam, UK), Alexa Fluor 647-conjugated anti-rabbit IgG (1:2000, ab150079, Abcam, UK), InsR monoclonal mouse antibody (1:50, ab983, Abcam, UK) or isotype control (1:50, ab91366, Abcam, UK), Alexa Fluor 488-conjugated anti-mouse IgG (1:1000, #4408, Cell signaling, USA), Alexa Fluor 488 conjugated InsR monoclonal goat antibody (1:50, FAB1544G, R&D Systems, USA) or isotype control (1:50, IC108G, R&D Systems, USA).
Antibodies used in Immunofluorescence analysis:
mouse InsR monoclonal antibody (1:50, sc-57342, Santa Cruz, USA), rabbit polyclonal antibody against p(Thr183/172)-AMPKα1/2 (1:100, YP0575, ImmunoWay, USA), Alexa Fluor 488-conjugated rabbit LDLR antibody (1:100, ab196377, Abcam, UK), CY3-conjugated goat anti-mouse secondary antibody (1:200, A10521, Thermofisher, USA), Alexa Fluor 647-conjugated goat anti-rabbit secondary antibody (1:200, A32728, Thermofisher, USA), rabbit IL-6 (1:100, GB11117, Servicebio, China), mouse TNF-α (1:100, GB11188, Servicebio, China) primary antibody, FITC-conjugated goat anti-mouse secondary antibody (1:100, A16079, Thermo fisher, USA), CY3-conjugated goat anti-rabbit secondary antibody (1:100, A10520, Thermo fisher, USA).

Validation

Each primary antibody has been validated based on the validation statements on the manufacturer's website.

Eukaryotic cell lines

Policy information about cell lines

Cell line source(s)	All cell lines used are obtained form ATCC or National Infrastructure of Cell Line Resource
Authentication	The identity of the cell line was authenticated with STR profiling (FBI, CODIS)
Mycoplasma contamination	The cell lines were checked free of mycoplasma contamination by PCR and culture.
Commonly misidentified lines (See ICLAC register)	None

Animals and other organisms

Policy information about studies involving animals; ARRIVE guidelines recommended for reporting animal research

Laboratory animals	Male C57BL/6J (6 weeks; 20-22 g)
Wild animals	The study did not involve wild animals.
Field-collected samples	The study did not involve samples collected from the field.
Ethics oversight	All experimental procedures were validated by the ethics committee of the institute of Materia medica, academy of medical sciences and Peking union medical college (Beijing, China).

Note that full information on the approval of the study protocol must also be provided in the manuscript.

Flow Cytometry

Plots

Confirm that:

☒ The axis labels state the marker and fluorochrome used (e.g. CD4-FITC).

☒ The axis scales are clearly visible. Include numbers along axes only for bottom left plot of group (a 'group' is an analysis of identical markers).

☒ All plots are contour plots with outliers or pseudocolor plots.

☒ A numerical value for number of cells or percentage (with statistics) is provided.

Methodology

Sample preparation	Please see Method sections:<br>Cellular uptake and P-gp mediated efflux of BBR-CTA-Mic in HepG2 cells;<br>Mechanism of BBR-CTA-Mic endocytosis;<br>In vitro pharmacological effect;<br>Flow cytometry analysis<br>and in supplementary information.
Instrument	BD FACSARIA III;<br>BD FAC SCalibur.
Software	FlowJo_V10
Cell population abundance	Firtsly cell clusters and debris are excluded by gating the FSC&SSC dimension, then the abundance of hepatocytes, kupper cells and endothelial cells are shown in the scatter plot of CD14-PE&F4/80-APC dimension, divided into 4 quadrants, among which the hepatocytes are located in double-negative quadrant, kupper cells in double-positive quadrant, and endothelial cells in single-positive quadrant.
Gating strategy	Kupper cells, endothelial cells and hepatocytes are gated in liver tissue cell suspensions by selecting F4/80+ populations, F4/80-/CD14+ populations and negative populations respectively.

☒ Tick this box to confirm that a figure exemplifying the gating strategy is provided in the Supplementary Information.
